# Swab and Send: a citizen science, antibiotic discovery project

**DOI:** 10.2144/fsoa-2020-0053

**Published:** 2020-05-06

**Authors:** Adam P Roberts

**Affiliations:** 1 Centre for Drugs and Diagnostics & Department of Tropical Disease Biology, Liverpool School of Tropical Medicine, Pembroke Place, Liverpool, L3 5QA, UK

**Keywords:** antibiotic, antifungal, citizen science, crowdsourcing, drug discovery

Antimicrobial resistance is one of the greatest global healthcare challenges of our time. It is an anthropogenically amplified natural response by bacteria and fungi to the selective pressure provided by extensive and excessive use of antimicrobials. The scale of use of antimicrobial compounds is truly astonishing and underpins their position as cornerstones of both developing and developed national-scale healthcare and food production systems.

While the importance of antimicrobials to society is in no doubt, companies involved in research and development of antimicrobials struggle to make their research effort viable from the perspective of economic return. There has been an exodus of pharmaceutical companies from the antimicrobial development field, including pharmaceutical giants such as, AstraZeneca (UK), Novartis (Switzerland) and Sanofi (France) and the recent bankruptcy of smaller companies (Achaogen, Melinta and Tetraphase), despite these having successfully navigated complex regulatory hurdles and bringing an antibiotic to market. The reasons for these divestitures and insolvencies are due to the lack of returns associated with the current for-profit economic model of financial reimbursement for bringing a new antimicrobial product to market [1].

While it is encouraging to see an increase in the number of compounds in pre-clinical testing, which has been catalysed by recent increased funding, there is still a recognised lack of compounds entering the pipeline [2]. Due to the lack of activity in the drug discovery space much of the early activity has fallen to academic groups around the world. Many of the antibiotics that we currently use in medicine are natural products produced by bacteria or fungi from soil. Finding microbes that produce antibacterial or antifungal natural products is not difficult, provided you have trained microbiologists and a well-appointed laboratory. However, it is time consuming and costly to do it at scale. Due to the low probability of any of these antimicrobial compounds being useful in medicine, there is also a relatively low amount of funding available for the initial discovery of products compared with later stage antimicrobial development projects.

In addition to the development of new antimicrobials, part of the recommended response to antimicrobial resistance is increased public awareness campaigns. A multitude of successful public-engagement projects have emerged over the last few years [3], in different formats ranging from the Surgeon X graphic novel to the Game Dr contemporary dance production; Antibiotic Apocalypse, and larger information campaigns such as the ‘Superbugs’ exhibition at the Science Museum in the UK and the World Antibiotic Awareness Week [4].

In order to expand initial antimicrobial discovery activity beyond soil microbes and to circumvent the absence of funding and create a dynamic, long-term, public-engagement activity, the citizen science project ‘Swab and Send’ was launched in early 2015. It was designed to enable individuals to decide where and what to sample in order to try and find bacteria or fungi, which can produce antimicrobial products against a range of bacterial and fungal indicator strains.

Citizen science is historically rooted in antimicrobial discovery. Indeed, the first strain of *Penicillium notatum*, the original producer of penicillin isolated by Alexander Fleming, was replaced with a strain of *Penicillium chrysogenum* producing many times more penicillin. This *P. chrysogenum* was isolated from a cantaloupe as part of a nationwide citizen science effort aimed at finding more efficient penicillin producers [5,6]. There are more recent citizen science projects aimed at looking for new antimicrobials produced by soil microbes, including ‘The Citizen Science Soil Collection Program*’* based at the University of Oklahoma (USA) [7], ‘Drugs from Dirt’ based at Rockefeller University (NY, USA) [8] and the ‘Antibiotics Unearthed*’* project run by the Microbiology Society in the UK [9], which was itself inspired by the Small World Initiative [10], a US based program that encourages students to pursue careers in science by focussing the reducing numbers of effective antibiotics.

Swab and Send relies on social media; primarily using Facebook to report results back to participants and Twitter (#swabandsend) to reach potential participants including members of the public, school, colleges and other organisations. As external research funding is rare for this initial discovery activity due to the high likelihood of failure, costs are covered by a small pledge from participants using an online form. Swabs are posted with instructions detailing what to consider when deciding what to sample. While imagination is encouraged in terms of what is sampled, we do say that animals and humans should not be swabbed for safety reasons, we do however regularly receive samples from humans (particularly school children) and many animals, with an inexplicable over-representation of cats.

Once we receive the samples back in the laboratory, they are streaked onto agar plates and incubated for 4–7 days at room temperature. Each individual morphologically distinct colony is then picked, using a sterile pipette tip, into a microtiter plate well containing growth medium and incubated at room temperature overnight. Following the phenotypic assays described below, the microtiter plates are stored at -80°C, following the addition of glycerol, for future assays.

The phenotypic assays are carried out by inoculating agar plates with an overnight culture of our indicator strains (currently *Micrococcus luteus*, *Staphylococcus aureus*, *Escherichia coli*, *Candida albicans* or *Candida auris*) and allowing to dry. The cultures from the microtiter plates are replica plated onto these pre-inoculated agar plates, which are incubated at room temperature for 2–3 days checking for growth each day. We analyse the assay plates for zones of inhibition around the colonies originating from the swabs and all the agar plates are photographed along with a template sheet detailing which sample is positioned where within the microtiter plate. These are uploaded onto the dedicated Facebook project webpage [11,12] with an explanation of the results, highlighting interesting hits we have found. Further updates are provided as the project moves forward, both in terms of results (e.g., we have recently included a post about a multidrug resistant *Klebsiella grimontii* strain isolated from a reusable water bottle [13]) and significant developments in the antibiotic resistance and chemotherapy. This serves as a permanent historical record of all the project activity since its launch and as a useful repository for important reports on antimicrobial resistance.

Currently, hits against *E. coli*, *C. albicans* and *C. auris* are taken forward to cell free assays to determine if we can follow the antimicrobial activity of the secreted molecules. The hit isolates are also genome sequenced for de-replication purposes as we want to avoid working on a compound already reported and candidate molecules can often be inferred from genomic data where we can identify biosynthetic gene clusters.

Feedback on the project is overwhelmingly positive and is left via the website and via personal correspondence to the author. It is clearly reaching a large number of individuals evidenced by analysis of over 2000 swabs and the testing of over 4500 isolates against our indicator strains. We have received swabs from numerous members of the public, more than 20 schools, colleges and universities and have taken part in multiple events including the Microbiology Society 75th Anniversary, the Surgeon X Launch Party, the TEDxLSTM talks, the Big Bang Fair, Thought Bubble Festival and New Scientist Live to name but a few. Coverage of the project has appeared in various National Newspapers in the UK including the Times, The Guardian and The Telegraph plus The Atlantic in the US and various other publications and radio broadcasts throughout the world. In addition, we have worked with the BBC in the UK on Trust Me I’m a Doctor and more recently on The Truth About Antibiotics. The reach of the project from the various media outlets and the social media platforms is substantial with some social media posts reaching in excess of 1 million views and upwards of 50,000 interactions.

Scientifically we are also finding interesting bacterial isolates that are being taken forward into traditional scientific investigations within the Centre for Drugs and Diagnostics at Liverpool School of Tropical Medicine (UK). We currently have approximately 50 hits against each of the *Candida* species, 60 against *E. coli*, 100 against methicillin-resistant *Staphylococcus aureus* and in excess of 500 against *M. luteus*. Excitingly we have isolates that can kill or inhibit the growth of different combinations of the indicator strains, including a small number active against only one of the two *Candida* species. This is likely to be advantageous when we are at a stage to determine the target of the compound(s) produced by these isolates.

In addition to the continuing public-engagement activities and the ongoing investigations of antimicrobial producing strains, the project has also resulted in a valuable physical resource. Along with what we have already screened, we have over 10,000 isolates stored, assay-ready, as glycerol stocks in microtiter plates, awaiting antimicrobial assays. The entire isolate library, which is possibly one of the most random microbial isolate libraries in the world, is available for screening should a project with a suitable assay present itself.

Looking at the future of antimicrobial discovery, which is constantly stated as being an international priority, it is not difficult to envisage nationally funded antimicrobial screening programmes of samples sent in by the public, which could be used to bolster national and international efforts to refill the antimicrobial pipeline. The major factors limiting success of screening projects is scale and capacity, which are dependent on resources, both financial and expertise. We would be wise to protect the latter by providing the former.

## References

[1] Singer AC, Kirchhelle C, Roberts AP (Inter)nationalising the antibiotic research and development pipeline. Lancet Infect. Dis. 20(2), e54–e62 (2020). 31753765 10.1016/S1473-3099(19)30552-3

[2] Butler MS, Paterson DL Antibiotics in the clinical pipeline in October 2019. J. Antibiotics (2020). 10.1038/s41429-020-0291-8 PMC7223789 32152527

[3] Redfern J, Bowater L, Coulthwaite L, Verran J Raising awareness of antimicrobial resistance among the general public in the UK: the role of public engagement activities. JAC-AMR 2(1), (2020) (Epub ahead of print). 10.1093/jacamr/dlaa012 PMC8210175 34222970

[4] WHO. World antibiotic awareness week (2020). www.who.int/news-room/campaigns/world-antibiotic-awareness-week

[5] Gaynes R The discovery of penicillin-new insights after more than 75 years of clinical use. Emerg. Infect. Dis. 23(5), 849–853 (2017).

[6] Laura Bowater The Microbes Fight Back: Antibiotic Resistance. 85 Royal Society of Chemistry, London, UK (2017).

[7] The University of Oklahoma. Citizen science soil collection program (2020). https://whatsinyourbackyard.org/participate/

[8] Drugs from dirt (2017). http://drugsfromdirt.org/DrugsFromDirt/

[9] Microbiology society – antibiotics unearthed (2020). https://microbiologysociety.org/members-outreach-resources/outreach-resources/antibiotics-unearthed.html

[10] Small World Initiative (2019). www.smallworldinitiative.org/about

[11] Swab and Send Facebook Page (2020). www.facebook.com/swabandsend/

[12] Swab and Send Project (2020). www.lstmed.ac.uk/public-engagement/swab-send

[13] Hubbard ATM, Newire E, Botelho J Isolation of an antimicrobial-resistant, biofilm-forming, *Klebsiella grimontii* isolate from a reusable water bottle. Microbiol. Open e1023 (2020) (Epub ahead of print). 10.1002/mbo3.1023 PMC7294305 32126585

